# Involvement of Carboxylesterase in Hydrolysis of Propranolol Prodrug during Permeation across Rat Skin

**DOI:** 10.3390/pharmaceutics5030371

**Published:** 2013-07-01

**Authors:** Teruko Imai, Yuko Takase, Harunobu Iwase, Mitsuru Hashimoto

**Affiliations:** Graduate School of Pharmaceutical Sciences, Kumamoto University, 5-1 Oe-honmachi, Chuo-ku, Kumamoto, 862-0973, Japan; E-Mails: ohurak@kumamoto-u.ac.jp (Y.T.); itakako1007@hotmail.com (H.I.); hashimot@cc.matsuyama-u.ac.jp (M.H.)

**Keywords:** prodrug, skin permeation, hydrolysis, carboxylesterase

## Abstract

The use of a prodrug, a conjugate of an active drug with a lipophilic substituent, is a good way of increasing the cutaneous absorption of a drug. However, the activity of dermal hydrolases has rarely been investigated in humans, or experimental animals. In the present study, we focused on the identification of rat dermal esterases and the hydrolysis of a prodrug during permeation across rat skin. We found that carboxylesterase (CES), especially the rat CES1 isozyme, Hydrolase A, is expressed in rat skin and that the hydrolysis of *p*-nitrophenyl acyl derivatives and caproyl-propranolol (PL) was 20-fold lower in the 9000*g* supernatant fraction of skin homogenate than in liver microsomes. A permeation study of caproyl-PL was performed in rat full-thickness and stripped skin using a flow-through diffusion cell. Caproyl-PL was easily partitioned into the stratum corneum and retained, not only in the stratum corneum, but also in viable epidermis and dermis. Caproyl-PL could barely be detected in the receptor fluid after application to either full-thickness or stripped skin. PL, derived from caproyl-PL, was, however, detected in receptor fluid after extensive hydrolysis of caproyl-PL in viable skin. Permeation of PL was markedly decreased under CES inhibition, indicating that the net flux of caproyl-PL is dependent on its conversion rate to PL.

## 1. Introduction

Skin is the largest organ of the body, its primary function being to act as a permeability barrier to the surrounding environment. The outer layer of the skin, the stratum corneum, has an essential role as a barrier to the transport of water and xenobiotics. However, any xenobiotics that do penetrate the skin are biotransformed into harmless or less harmful compounds by various enzymes in the epidermis and dermis. Cutaneous metabolism introduces new chemicals into the systemic circulation and is an important determinant in the penetration of many compounds through the skin [1]. Polar metabolites are more able to, than non-polar compounds, penetrate an aqueous environment such as the dermis and viable epidermis. A wide range of Phase I and Phase II metabolic biotransformations are able to be carried out in the skin, and most dermal metabolism occurs in basal keratinocytes in the epidermis [2]. Constitutive expression of xenobiotic-metabolizing enzymes has been detected in normal human keratinocytes [3].

Prodrugs, which are designed to improve the bioavailability and/or toxicity of parent drugs, are generally modified by ester linkage. Prodrugs are more easily partitioned into the stratum corneum by diffusion due to their high lipophilicity. Some prodrugs are transported into the blood as original prodrugs, but others are subcutaneously hydrolyzed to polar parent drugs by esterases in the viable epidermis underlying the stratum corneum. Polar compounds permeate the dermis and viable epidermis more easily than non-polar compounds, and the penetration rate of prodrugs is controlled by cutaneous hydrolysis. Therefore, both the partition of a prodrug into the stratum corneum and its cutaneous hydrolysis are key factors in the overall permeation of a prodrug through the skin. 

The specific activity of dermal enzymes is much lower than their hepatic counterparts. However, because of the retention of lipophylic prodrugs in viable epidermis, they are extensively metabolized in the skin. Hewitt *et al.* [1] showed that esters applied on the surface of the skin were completely hydrolyzed during dermal absorption using an *in vitro* diffusion cell. We have previously reported that the ester derivatives of propranolol (PL) are hydrolyzed during permeation through hairless mouse skin, and their hydrolysis rates are positively related with their hydrophobicities [4].

It has been demonstrated that several esterases such as carboxylesterase (CES; EC 3.1.1.1) and arylesterase, present in human and rat skin, are responsible for the biotransformation of prodrugs [5]. In addition, we have previously reported that the hydrolysis of PL ester derivatives is catalyzed by CES and butyrylcholinesterase in hairless mouse skin [6]. 

CESs are member of the α/β hydrolase fold family and show ubiquitous tissue expression profiles with high levels in liver, small intestine, and lung [7,8,9]. The mammalian CESs comprise a multigene family, and isozymes are classified into five main CES families (CES1–CES5), and several subfamilies, according to the homology of their amino acid sequences [10]. The majority of CESs involved in detoxification of xenobiotics are from the CES1 and CES2 families. Mammalian CES1 isozymes are highly expressed in most organs, while CES2 isozymes are expressed in a limited number of organs, including intestine, liver, and kidney [7,11]. It has been reported that human skin expresses mainly CES1 isozyme (hCE1), and to a lesser extent CES2 isozyme (hCE2) [5], while Zhu *et al.* [12], reported a high expression of hCE2 and a weak expression of hCE1 in human HaCaT keratinocytes. Unfortunately, esterases, including CESs, are rarely investigated in animal skin.

In the present study, we have focused on the identification of rat dermal esterases, especially CES isozymes. The hairless mouse and minipig are commonly used for percutaneous experiments, but their CES isozymes have hardly been investigated. In comparison, rat CES isozymes have been more extensively analyzed, so we investigated the relation between dermal hydrolysis and CES expression in rat skin. We selected caproyl-PL as a model hydrophobic prodrug, and demonstrated the effect of hydrolysis during prodrug permeation across rat skin. 

## 2. Materials and Methods

### 2.1. Materials

Racemic *O*-caproyl-PL hydrochloride was synthesized from PL hydrochloride (Wako Pure Chemical Industries, Ltd., Osaka, Japan) and caproyl chlorides (Tokyo Kasei, Tokyo, Japan) according to a previously described method [13]. *p*-Nitrophenol, *p*-nitrophenylacetate (PNPA), *p*-nitrophenylbutyrate (PNPB), and bis-*p*-nitrophenyl phosphate (BNPP) were purchased from Nacalai Tesque, Inc. (Kyoto, Japan). All other chemicals and reagents were of analytical or biochemical grade.

### 2.2. Animals

Male Wistar rats (8 weeks, 240–260 g) were used after overnight fasting with free access to water. All animal experimental protocols were approved by the Ethics Review Committee for Animal Experimentation of Kumamoto University.

### 2.3. Preparation of Skin Homogenate

After hair was carefully clipped and shaved, rats were sacrificed by exsanguination under ether anesthesia. Abdominal and dorsal skin was removed using a scalpel and dissection scissors. A few pieces of skin were stored in liquid nitrogen for extraction of total RNA. The skin was minced, mixed with five volumes of ice-cold 4-(2-hydroxyethyl)-1-piperazineethanesulfonic acid (HEPES) buffer (50 mM, pH 7.4) containing 0.15 M KCl, and homogenized using a Polytron (Kinematica, Lucerne, Switzerland) on ice. Whole homogenate was filtered through a funnel containing buffer-soaked cotton wool. After centrifugation of the skin homogenate at 9000*g* for 20 min at 4 °C, the supernatant fraction (S9) was obtained. Protein content was determined by the method described by Bradford [14] using bovine serum albumin (BSA) as standard. The skin S9 was stored at −80 °C until use.

### 2.4. Preparation of Homogenates of Liver, Kidney, and Testis

After rats were sacrificed by exsanguination under ether anesthesia, a cannula was placed in the inferior vena cava, and the liver and kidney were perfused with ice-cold 0.15 M KCl to remove blood. The excised liver, kidney, and testis were washed with ice-cold 0.15 M KCl, and finely minced using scissors. Minced tissues were homogenized with 3 volumes of 50 mM HEPES buffer (pH 7.4) containing 0.15 M KCl in a Potter–Elvehjem glass homogenizer equipped with a Teflon pestle under ice-cold conditions. Homogenates (25% wet *w*/*v*) were centrifuged at 9000*g* for 20 min at 4 °C, to give the S9 fraction. S9 fraction was further centrifuged at 100,000*g* for 1 h at 4 °C. The resulting pellets were resuspended in 50 mM HEPES buffer (pH 7.4) containing 0.15 M KCl to prepare the microsomal fraction. Protein concentrations were determined by the method of Bradford [14] using BSA as standard. S9 and the microsomal fraction were stored at −80 °C until use.

### 2.5. Native Polyacrylamide Gel Electrophoresis (PAGE)

Polyacrylamide gel electrophoresis (PAGE) was performed as described by Mentlein *et al.* [15]. Polyacrylamide gels (7.5% *w*/*w*), containing 1% (*w*/*v*) nonidet P-40 for solubilization of proteins, were used for the separation of native enzymes. After electrophoresis of tissue samples (10–40 μg protein), the gels were stained for esterase activity with 1-naphthylacetate through coupling to liberated 1-naphthol with Fast Red TR-salt.

### 2.6. Total RNA Preparation for Tissue and Reverse Transcription (RT)-Polymerase Chain Reaction (PCR)

Total RNA was extracted from skin, liver, kidney, and testis using ISOGEN (Nippon Gene Co. Ltd., Toyama, Japan). To prevent contamination with genomic DNA, the extracts were treated with DNase I (Invitrogen, Carlsbad, CA, USA). RNA concentration and purity were determined spectrophotometrically. Total RNA (2 μg) was reverse-transcribed using 100 pmol Oligo (dT) prime, 1 mM dNTP, and RNase H free ReverTra Ace (Toyobo, Osaka, Japan) with one cycle of the RT reaction (42 °C for 60 min). After digestion of the remaining RNA with RNase H, the reverse transcription samples were subjected to subsequence PCR using platinum TaqDNA polymerase (Invitrogen, Carlsbad, CA, USA). Specific primers for rat CES isozymes were as follows: Hydrolase A (X51974) sense: 5'-CTGGACTTACTTGGAAACCC-3' and antisense: 5'-TGCAACCAAGTCCTGGAACA-3'; Hydrolase B/C (X81825/U10697) sense: 5'-CCAAAGACCCAAGGATGTAG-3' and antisense: 5'-TGAGGTTGTCTCTTAGCCAG-3'; RL2 (X81395) sense: 5'-ACACAGATGACCCAGACAGA-3' and antisense: 5'-CAGTGGCTTCATAGCCAGAA-3'; GAPDH (NM_017008) sense: 5'-ACCACAGTCCATGCCATCAC-3' and antisense: 5'-TCCACCACCCTGTTGCTGTA-3'.

### 2.7. Hydrolysis Experiments

Hydrolysis of PNPA, PNPB, and caproyl-PL were performed in S9 and microsomal fractions of rat liver, testis, kidney and skin. Tissue samples were diluted with 50 mM HEPES (pH 7.4) buffer at appropriate protein concentrations and preincubated for 5 min at 37 °C. The hydrolysis reaction was initiated by the addition of test compounds dissolved in dimethyl sulfoxide (DMSO). The final concentration of DMSO was less than 0.5%, which has no effect on hydrolytic activity. The rate of hydrolysis of PNPA and PNPB (final concentrations 15–500 μM) was determined by the initial linear increase in absorbance of *p*-nitrophenol at 405 nm (V-530, JASCO Co., Tokyo, Japan). 

For the hydrolysis of caproyl-PL, the reaction was terminated by addition of 5 mL ethylacetate. After extraction of PL, as previously described [16], stereoselective determination was carried out using high-performance liquid chromatography (HPLC). 

### 2.8. *In Vitro* Skin Permeation Study

**Figure 1:**
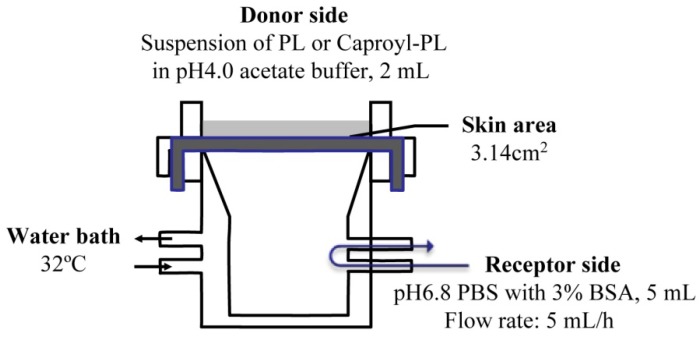
Scheme of transdermal permeation experiment using a flow-through diffusion cell.

The *in vitro* percutaneous penetration study was performed using a flow-through diffusion cell at 32 °C, as shown in Figure 1. The receiving chamber had a volume of 5 mL and the skin area available for diffusion was 3.14 cm^2^. Fresh excised abdominal skin was used for these experiments. To strip skin, stratum corneum was removed by 20 successive strippings using cellophane adhesive tape (Nichiban K.K., Japan) according to conventional methods. The excised skin was mounted between half-cells with the dermal side in contact with receptor fluid (pH 6.8 phosphate-buffered saline (PBS) with 3% BSA). Receptor fluid flow rate was 5 mL/h. Two milliliters of drug suspension (drug amount = twice the amount required for saturation) in pH 4.0 acetate buffer was added to the donor chamber. 

Nonenzymatic hydrolysis of caproyl-PL to PL was observed in buffer. This hydrolysis was <0.1% in 12 h under the most stable conditions of caproyl-PL (pH 4.0 acetate buffer). Hydrolysis was greater under basic buffer conditions (pH 6.8). However, the addition of BSA to the receptor fluid effectively prevented the hydrolysis of caproyl-PL. The hydrolysis of caproyl-PL was <0.05% during the sampling period (1 h) at pH 6.8 in PBS containing 3% BSA.

In an inhibition study for CES, skin mounted between the half-cells was preincubated with the dermal side in contact with 2 mM BNPP (dissolved in pH 6.8 PBS containing 3% BSA) for 1 h.

### 2.9. HPLC Assay

The HPLC system comprised a JASCO PU-980 pump, a JASCO 980-UV detector, a JASCO AS950 autosampler, a JASCO CO-965 column oven and a JASCO 1520S fluorescence detector (JASCO Co., Tokyo, Japan), and a Shimadzu chromatopac C-R7A plus (Shimadzu Co., Ltd., Kyoto, Japan). PL formation in the hydrolysis experiment was stereoselectively determined using Chiralcell OD column (10 μm, 250 × 4 mm inner diameter (i.d.); Daicel Chemical Industries, Ltd., Tokyo, Japan) with a mobile phase of hexane/2-propanol (9:1, *v*/*v*) at a flow rate of 1.0 mL/min. For the skin penetration experiment, LiChrosorb RP-select B column (7 μm, 250 × 4 mm i.d.; Merck Ltd., Tokyo, Japan) was used with a mobile phase of acetonitril/20 mM KH_2_PO_4_ (1:1 *v*/*v*) at a flow rate of 1.0 mL/min. PL and caproyl-PL were detected with excitation and emission wavelengths of 285 and 340 nm, respectively. The quantitative limitation was 30 pmol for PL and 60 pmol for caproyl-PL as injected amounts. 

### 2.10. Data Analysis

The *in vitro* permeation parameters were calculated from the penetration data using the following equations: *J*_s_ = (*K*_m_*DC*)/δ = *K*_p_*C* and τ = δ^2^/6*D*, where *J*_s_ is steady state flux, *K*_m_ is the solvent membrane partition coefficient of drug, *D* is diffusion coefficient, *C* is drug concentration in donor chamber, δ is thickness of rat skin, *K*_p_ is the permeability coefficient of drug, and τ represents lag time.

## 3. Results

### 3.1. Detection of Esterase Activity of Rat Tissues on Native PAGE Gel

**Figure 2:**
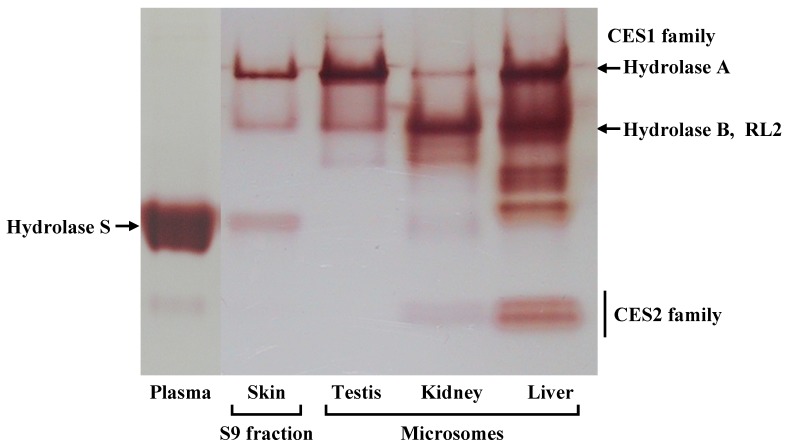
Electrophoretic pattern of esterase in several rat tissues. 1-Naphthol, a dye derived from hydrolysis of 1-naphthylacetate, was used to detect esterases in polyacrylamide gelelectrophoresis (PAGE) through the formation of an insoluble complex with Fast Red TR. The quantities of skin S9 and tissue microsomal proteins loaded were 40 and 10 μg, respectively. Arrows indicate the bands corresponding to each carboxylesterase 1 (CES1) isozyme.

Figure 2 shows a native PAGE gel, stained for esterase activity by 1-naphthylacetate. All proteins with hydrolytic activity can be visualized by this technique. Three bands were detected in rat skin S9. Formation of these bands was not inhibited by the addition of ethopropazine, a specific inhibitor of butyrylcholinesterase [17], or EDTA, an inhibitor of paraoxonase [17]. In order to compare skin esterase with tissue-specific CES isozymes, the microsomal fraction of each rat organ was applied to a native PAGE gel. As CESs are present in endoplasmic reticulum (ER) through binding with the KDEL receptor on the ER membrane, CES activity is high in the microsomal fraction. The upper strong band of skin S9 shows the same migration as the major band of testis microsomes. The second weak band is at the same position as the major band in rat kidney microsomes. It has been reported that the major esterases of rat testis and kidney are Hydrolase A and Hydrolase B, respectively [18,19]. Both Hydrolase A and Hydrolase B belong to the CES1 family and have similar molecular weights (around 60 kDa) and isoelectric points (pI 6.0 and 6.5, respectively). However, their migration rates are different as Hydrolase A is a trimer and Hydrolase B is a monomer. The weak band migrating in the center of the gel is at the same position as the major band in rat plasma. In rat plasma, only one major band was observed corresponding to the CES1 isozyme, Hydrolase S [17,20]. Rat liver microsomes showed several bands representing CES1 and CES2 enzymes. CES2 enzymes were located in the lower part of the gel, because of their lower pI value (around 5.0) and their monomeric form [21]. In contrast to liver microsomes, skin S9 did not show any band corresponding to CES2 isozymes.

### 3.2. Expression of mRNA Level of Carboxylesterase *(CES)* Isozymes

**Figure 3:**
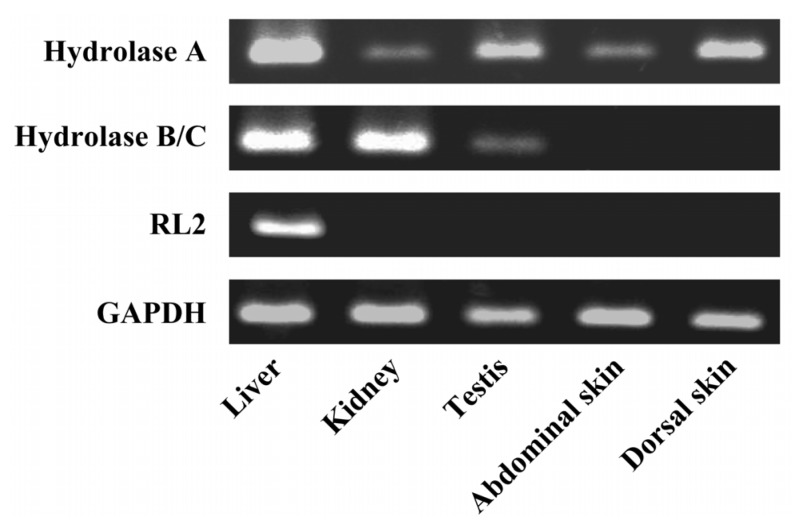
The mRNA expression levels of rat *CES1* isozymes in rat liver, kidney, testis and skin. The gene expression levels of rat *CES1* isozymes and *GAPDH* were assessed by semiquantitative RT-PCR.

We measured the mRNA levels of 4 *CES1* isozymes, *Hydrolase A*, *Hydrolase B*, *Hydrolase C*, and *RL2*, in several tissues. In terms of amino acid sequence, *Hydrolase B* is very similar to *Hydrolase C* (93% homology). Therefore, the expression of *Hydrolase B/C* mRNA was measured using primer designed from identical nucleotide sequences. Figure 3 shows the mRNA expression levels of *CES1* isozymes. mRNAs for all *CES1* isozymes were present in rat liver, while testis and kidney expressed predominantly mRNAs from *Hydrolase A* and *Hydrolase B/C*. Among the CES1 family, only Hydrolase A mRNA was expressed in both abdominal and dorsal skin. As expected from native PAGE, the expression of *CES2* isozymes was less than the limit of detection (data not shown).

### 3.3. Hydrolase Activity of Rat Tissues

In order to evaluate cutaneous hydrolase activity, the hydrolysis rates of PNPA, PNPB, and caproyl-PL were determined in skin S9 fraction, and compared with those in rat liver, testis, and kidney. Since PNPA and PNPB are substrates for not only CES but also various esterases, their hydrolysis represents the total hydrolase activity of each tissue. Caproyl-PL is a good substrate for most CES isozymes in various animals [21]. It is enantioselectively hydrolyzed by rat CES1 isozymes, while it is non-enantioselectively hydrolyzed by rat CES2 isozymes [21]. 

The *K*_m_ and *V*_max_ of hydrolysis of PNPA and PNPB are given in Table 1. The *K*_m_ values for PNPA and PNPB in rat skin were nearly the same, and similar to those of testis microsomes. However, kidney microsomes, expressing mainly Hydrolase B, showed a 2.6-fold greater *K*_m_ value for PNPA than PNPB. The *V*_max_ for PNPA in skin S9 was 19-fold smaller than in liver microsomes, while the *V*_max_ for PNPB was 9-fold smaller than in liver microsomes. Additionally, the *V*_max_/*K*_m_ values, representing intrinsic clearance, were 21-fold and 24-fold smaller for PNPA and PNPB, respectively, in rat skin S9 than in liver microsomes. 

**Figure 4:**
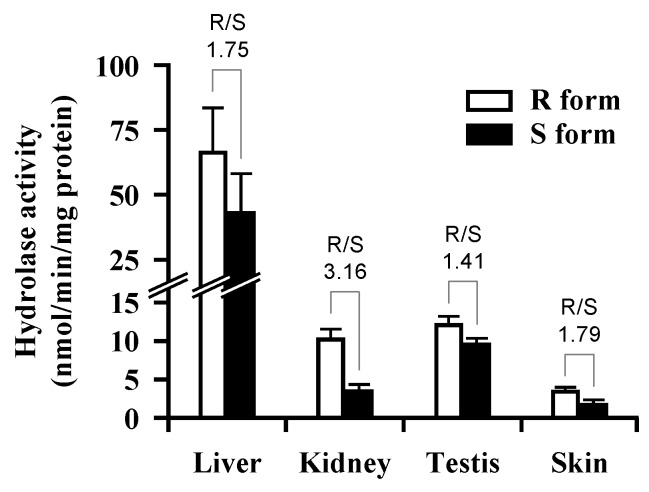
Formation of *R*- and *S*-PL by hydrolysis of racemic caproyl-PL in S9 fraction of several rat tissues. Racemic caproyl-PL (100 μM) was hydrolyzed in the S9 fraction from several rat tissues diluted with 50 mM HEPES buffer (pH 7.4). Open and filled columns show the hydrolase activity for R and S forms, respectively. Each value represents the mean ± S.D. (*n* = 3).

Figure 4 shows the hydrolysis rate of caproyl-PL in rat tissue S9. In S9 fractions from all tissues, the *R*-isomer of caproyl-PL was more rapidly hydrolyzed than the *S*-isomer, since CES1 isozymes are in the majority in these tissues. In skin and testis, both of which contain Hydrolase A as the major esterase, the *R*/*S* ratio was 1.79 and 1.41, respectively, similar to the 1.75 for liver S9. However, a *R*/*S* ratio of 3.16 was observed in kidney S9, much higher than in other tissues due to the expression of Hydrolase B. For caproyl-PL in rat skin S9, the *K*_m_ value was 19.7 ± 5.36 μM and *V*_max_ was 9.24 ± 2.95 nmol/min/mg S9 protein.

**Table 1:** Kinetic parameter for hydrolysis of PNPA and PNPB in rat skin S9, testis, kidney, and liver microsomes.

	Skin	Testis	Kidney	Liver
	S9	microsomes	microsomes	microsomes
**PNPA**				
*K*_m_ (μM)	55.5 ± 8.18	34.6 ± 1.27	268 ± 83.8	57.0 ± 18.2
*V*_max_ (nmol/min/mg protein)	188 ± 30.3	1,606 ± 333	2,432 ± 497	3,570 ± 810
*V*_max_/*K*_m_ (mL/min/mg protein)	3.39 ± 0.332	46.3 ± 7.99	9.59 ± 3.05	72.0 ± 44.1
**PNPB**				
*K*_m_ (μM)	97.1 ± 30.2	63.4 ± 13.9	97.0 ± 16.6	37.1 ± 9.42
*V*_max_ (nmol/min/mg protein)	460 ± 127	2,978 ± 398	994 ± 286	4,220 ± 521
*V*_max_/*K*_m_ (mL/min/mg protein)	4.77 ± 0.268	49.2 ± 15.1	10.2 ± 1.55	117 ± 19.7

PNPA (25–500 μM) and PNPB (15–500 μM) were incubated with skin S9 (300 μg/mL), testis microsomes (10 μg/mL for PNPA, 5 μg/mL for PNPB), kidney microsomes (10 μg/mL), and liver microsomes (20 μg/mL for PNPA, 10 μg/mL for PNPB) diluted in 50 mM HEPES buffer (pH 7.4). Each value represents the mean ± S.D. (*n* = 3/4).

### 3.4. Permeation of Caproyl-PL through Full-Thickness Skin

**Figure 5:**
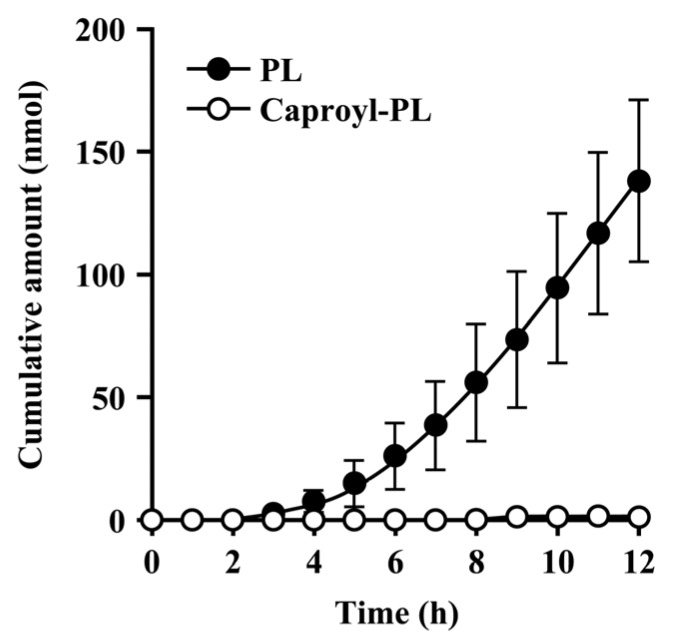
Permeation profile of caproyl-PL and PL across rat full-thickness skin after application of caproyl-PL. Open and closed symbols show caproyl-PL and PL transported into the receiver side, respectively. Each value represents the mean ± S.D. (*n* = 6).

A suspension of caproyl-PL in pH 4.0 acetate buffer was added to the donor compartment, to result in a constant concentration of 6.4 mM [4] throughout the permeation experiment. After penetration of caproyl-PL through rat full-thickness skin, both caproyl-PL and its hydrolysate, PL, were transported into the receptor fluid. Figure 5 shows periodical measurements of caproyl-PL and PL in receptor fluid. Interestingly, caproyl-PL was hardly found in receptor fluid, while PL was found in increasing concentrations. These data indicate the extensive hydrolysis of caproyl-PL and rapid diffusion of PL in viable skin. The permeation parameters are listed in Table 2. The skin concentration of caproyl-PL, 12 h after dosing, showed 1900 ± 630 nmol/g tissue, that is 3.5-fold higher than the value for PL (550 ± 120 nmol/g tissue). Thus, in spite of the high concentrations of caproyl-PL, it did not permeate through rat full-thickness skin, suggesting that it is retained in the skin. The *K*_p_ value calculated from total concentrations of caproyl-PL and PL was 3.1 cm/h, and the observed lag-time was 4.9 h. These data indicate that caproyl-PL is easily taken up, and retained, by skin where it is hydrolyzed to PL, a hydrophilic and permeable compound in viable epidermis.

**Table 2:** Pharmacokinetic parameters for permeation of caproyl-PL across rat skin.

	Full-thickness skin			Stripped skin			Full-thickness skin with BNPP		
	Caproyl-PL	PL	Total	Caproyl-PL	PL	Total	Caproyl-PL	PL	Total
Permeated amount after 12 h (nmol)	1.7 ± 1.4	140 ± 35	140 ± 33	12 ± 13	190 ± 55	200 ± 50	3.9 ± 3.4	21 ± 18	25 ± 21
Steady state flux (nmol·h^−1^)	0.40 ± 0.28	20 ± 6.2	20 ± 6.1	2.4 ± 0.98	22 ± 6.4	23 ± 5.5	0.83 ± 0.65	3.5 ± 2.6	4.1 ± 3.1
Lag time (h)	7.0 ± 0.78	4.9 ± 1.1	4.9 ± 1.1	4.7 ± 1.7	3.6 ± 0.38	3.6 ± 0.43	8.3 ± 1.2	6.3 ± 0.93	6.4 ± 0.92
*K*p (cm·h^−1^)	0.062 ± 0.044	-	3.1 ± 0.95	0.37 ± 0.15	-	3.6 ± 0.86	0.13 ± 0.10	-	0.64 ± 0.48
Skin concentration at 12 h (nmol/g tissue)	1,900 ± 630	550 ± 120	2,400 ± 780	3,200 ± 580	870 ± 44	4,100 ± 600	2,100 ± 220	130 ± 7.7	2,200 ± 230

Each value represents the mean ± S.D. (*n* = 6).

### 3.5. Permeation of PL through Full-Thickness and Stripped Skin

**Figure 6:**
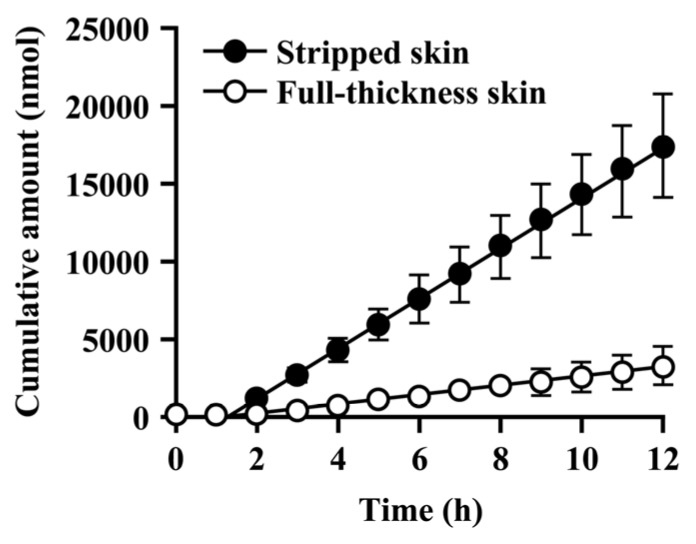
Permeation profile of PL across stripped skin and full-thickness skin after application of PL. Open and closed symbols show the amount of PL transported into the receiver side across full-thickness and stripped skin, respectively. Each value represents the mean ± S.D. (*n* = 6).

In order to study the permeation of PL, derived from caproyl-PL in viable skin, a permeation study was also performed using stripped skin. The permeation–time profile of PL is shown in Figure 6 and its kinetic parameters are given in Table 3. As shown in Figure 6, PL permeated more rapidly across stripped skin than across full-thickness skin. The steady-state flux of PL was 1600 ± 310 nmol/h in stripped skin, *i.e.*, 6.7-fold greater than in full-thickness skin. Additionally, the *K*_p_ value for PL was markedly greater in stripped skin than in full-thickness skin. These data indicate that the stratum corneum is the predominant barrier to the penetration of PL into skin, and that PL easily diffuses across viable skin. 

**Table 3:** Pharmacokinetic parameters for permeation of PL across rat skin.

	Full-thickness skin	Stripped skin	Full-thickness skin with BNPP
Amount permeated after 12 h (nmol)	2,600 ± 970	17,000 ± 3,600	2,600 ± 990
Steady state flux (nmol·h^−1^)	240 ± 94	1,600 ± 310	240 ± 87
Lag time (h)	1.5 ± 0.56	1.4 ± 0.36	0.84 ± 0.24
*K*_p_ (cm·h^−1^)	0.61 ± 0.24	4.2 ± 0.80	0.60 ± 0.22
Skin concentration at 12 h (nmol/g tissue)	9,400 ± 380	6,900 ± 3,700	9,900 ± 590

Each value represents the mean ± S.D. (*n* = 6).

### 3.6. Permeation of Caproyl-PL across Stripped Skin

Caproyl-PL was retained in full-thickness skin after partition into the stratum corneum. Generally, a hydrophobic compound has difficulty permeating into viable epidermis and dermis. In order to investigate the permeation of caproyl-PL in viable epidermis and dermis, a permeation study was performed using stripped skin. The permeation parameters of caproyl-PL across stripped skin are listed in Table 2. The steady-state flux and *K*_p_ values of intact caproyl-PL were 6-fold greater than in full-thickness skin, but their absolute values were still low. The skin concentration of caproyl-PL was slightly increased from 1900 ± 630 to 3200 ± 580 nmol/g tissue. The total concentrations of caproyl-PL and PL were nearly the same in stripped skin and full-thickness skin. These data indicate that the stratum corneum is not a barrier to the penetration of caproyl-PL into skin, and that caproyl-PL may be retained not only in the stratum corneum but also in the epidermis and dermis. 

### 3.7. Permeation of Caproyl-PL across Full-Thickness Skin under CES-Inhibition

The permeability of caproyl-PL was investigated under conditions in which the activity of hydrolyzing enzymes in the skin was reduced by inhibition of CES enzymes with BNPP. Cutaneous CESs were inhibited by preloading with 2 mM BNPP on the receiver side for one hour. BNPP was not added to the donor side in order to maintain normal conditions for the stratum corneum. The effect of BNPP on skin was first ascertained by examining the permeation of PL. No damage was observed visually and most parameters for permeation of PL, including steady state flux and skin concentration, were almost the same as with non-treated full-thickness skin (see Table 3). Only the lag time was found to be shorter under BNPP treatment, but this difference was not significant. These data indicate that treatment with BNPP hardly affects skin properties.

The permeation parameters of caproyl-PL after BNPP treatment of skin are given in Table 2. PL conversion from caproyl-PL was markedly decreased after treatment with BNPP. Even under hydrolysis-inhibited conditions, caproyl-PL was hardly transported into receiver fluid. The permeated amount, steady state flux and *K*_p_ value of intact caproyl-PL were only increased 2-fold by treatment with BNPP; in other words, these parameters were not significantly altered by BNPP treatment. The cutaneous concentration of caproyl-PL was also the same with and without BNPP treatment. These data indicate that caproyl-PL is retained in rat skin, independent of its hydrolysis to PL, and that a decrease in the activity of hydrolyzing enzymes leads to a decrease in the net transport of caproyl-PL.

## 4. Discussion

The barrier function of the skin to the absorption of active drugs resides in the outer-most layer, the stratum corneum. The use of a prodrug, a conjugate of active drug with a lipophilic molecule, is a way to achieve or increase cutaneous absorption. Conjugation via an ester bond is a conventional molecular modification. However, lipophilic drugs are retained in the skin. Therefore the conversion of a lipophilic prodrug to the active drug should occur at an appropriate rate in viable epidermis in order to obtain cutaneous activity and/or increase absorption of active drug into the bloodstream. CES is an abundant hydrolase in most organs [10]. There are at least four CES1 isozymes in the rat CES1 family, Hydrolase A, Hydrolase B, Hydrolase C, and RL2 [17,18,22]. In this study, we found Hydrolase A to be the predominant esterase in rat skin. In native PAGE, although a secondary band migrated to the same position as Hydrolase B, this band probably represents a monomer of Hydrolase A. The result of the RT-PCR also supports this explanation. The dissociation of trimeric CES1 isozyme has occasionally been found even in human liver samples [23]. Of these four CES1 isozymes, Hydrolase A shows the closest homology to hCE1 (78% homology) [10]. It has been reported that human skin expresses *hCE1* [5]. hCE1 hydrolyzes a wide variety of substrates, and its expression is utilized in the hydrolysis of various prodrugs to the active hydrophilic parent drugs [8,21].

For the hydrolysis of PNPA and PNPB, microsomes from testis, which expressed Hydrolase A, showed lower *K*_m_ values for PNPA than PNPB, while in kidney microsomes, which are abundant in Hydrolase B, the values for PNPB were lower than those for PNPA. Skin S9 showed similar properties to testis microsomes in its expression of Hydrolase A. The hydrolysis of PNPA and PNPB in skin S9 was around 20-fold slower than in liver microsomes. Caproyl-PL, a specific CES substrate, was also 20-fold more slowly hydrolyzed in skin S9 than in liver microsomes. As the CES content is higher in microsomes than in the S9 fraction, due to the localization of CES in the ER, these results indicate that the expression level of CES is sufficient to allow the hydrolysis of hydrophobic prodrugs during permeation through skin.

Since the hydrolysis of caproyl-PL by CES proceeds in the ER of viable epidermis and dermis, its rate of hydrolysis during cutaneous permeation depends on its dermal concentration. However, caproyl-PL was hydrolyzed to PL at nearly the same rate in both full-thickness and stripped skin. The concentration of caproyl-PL was 3200 ± 580 nmol/g tissue in stripped skin; this concentration is 160-fold higher than the *K*_m_ value (19.7 ± 5.36 μM), suggesting that saturation of hydrolysis occurs in stripped skin. These data led us to expect that hydrolysis would also be saturated in full-thickness skin. We therefore estimated the concentration of caproyl-PL in viable epidermis and dermis after its application to full-thickness skin. We assumed that the distribution of caproyl-PL in viable epidermis and dermis would be homogeneous, and that it would move from viable skin to receptor fluid by simple diffusion. From the steady-state flux of caproyl-PL (2.4 ± 0.98 nmol/h) and its cutaneous concentration in stripped skin, the permeation clearance from viable skin to receptor fluid was calculated as 0.75 mg tissue/h. Since steady-state flux was 0.37 ± 0.15 nmol/h in full-thickness skin, the concentration of caproyl-PL was predicted to be about 500 nmol/g tissue in viable epidermis and dermis. This concentration is 25-fold higher than the *K*_m_ value, suggesting saturation of cutaneous hydrolysis of caproyl-PL. The concentration of caproyl-PL in the stratum corneum can be calculated by subtraction from the 1900 ± 630 nmol/g tissue concentration of caproyl-PL in the skin. We therefore concluded that caproyl-PL applied to full-thickness skin is partitioned into the stratum corneum, where 75% of the dose is retained, only 25% of the dose being transferred into viable skin; this percentage is then hydrolyzed at maximum rate.

When CES-mediated hydrolysis was inhibited by BNPP, conversion to PL was markedly slower and there was a significant decrease in the net transport of caproyl-PL into receptor fluid. This clearly demonstrates that the net flux of prodrug is dependent on its conversion rate to the hydrophilic active drug. 

In this study, caproyl-PL was retained not only in the stratum corneum but also in viable epidermis and dermis. Caproyl-PL has a molecular weight of 358, a pKa of 9.1, and a log PC between 1-octanol and pH 4.0 phosphate buffer of 2.3, while PL has a molecular weight of 259, pKa of 9.44 and log PC of 0.38. Molecular size and basicity are important factors in determining skin permeability, in addition to hydrophobicity. The maximum size of molecule able to diffuse through the epidermis and dermis is smaller for human skin than rat skin due to differences in their relative thickness. Therefore, caproyl-PL is retained for longer in human skin than in rat skin. As caproyl-PL is more rapidly hydrolyzed by hCE1 than Hydrolase A [21], the steady-state flux of PL may be greater in human skin than in rat skin.

In the development of dermally applied prodrugs, there are two different aims, to obtain dermal activity and to obtain systemic activity. For dermal activity, prodrugs should be retained in whole skin where they are hydrolyzed to active drugs in a sustained manner. In this respect, caproyl-PL showed desirable properties as a prodrug. In order to obtain a systemic pharmacological activity, the intact prodrug should either rapidly diffuse into the bloodstream or be rapidly hydrolyzed in the skin. It is difficult to obtain rapid diffusion of hydrophobic prodrugs. Therefore, rapid conversion to active drug during penetration of a prodrug is obtained by selecting the optimum structure for recognition by hCE1. 

## 5. Conclusions

It has been demonstrated that CES is expressed in rat skin, as well as in other organs. The rat dermal CES is the CES1 isozyme, Hydrolase A. Among the four rat CES1 isozymes, Hydrolase A is the closest to the major human CES1 isozyme, hCE1. The expression of Hydrolase A in rat skin enables the dermal hydrolysis of prodrugs. The prodrug caproyl-PL was almost completely hydrolyzed by Hydrolase A during its permeation across rat skin. To enable the retention and sustained release of active drug in the skin, a dermal prodrug must have a relatively large molecular size and be hydrophobic, allowing slow diffusion in the skin and hydrolysis by the CES1 isozyme. However, no experimental animals have CES1 isozymes with the same substrate specificity as hCE1. Therefore, it is important to develop a prediction method for human cutaneous permeability from the physicochemical properties of prodrugs and their *in vitro* hydrolysis data.
